# Integrated metabolome and transcriptome analysis identifies candidate genes involved in triterpenoid saponin biosynthesis in leaves of *Centella asiatica* (L.) Urban

**DOI:** 10.3389/fpls.2023.1295186

**Published:** 2024-01-12

**Authors:** Lingyun Wan, Qiulan Huang, Cui Li, Haixia Yu, Guiyu Tan, Shugen Wei, Ahmed H. El-Sappah, Suren Sooranna, Kun Zhang, Limei Pan, Zhanjiang Zhang, Ming Lei

**Affiliations:** ^1^Guangxi Key Laboratory for High-Quality Formation and Utilization of Dao-Di Herbs, Guangxi Botanical Garden of Medicinal Plants, Nanning, China; ^2^National Center for Traditional Chinese Medicine (TCM) Inheritance and Innovation, Guangxi Botanical Garden of Medicinal Plants, Nanning, China; ^3^National Engineering Research Center for the Development of Southwestern Endangered Medicinal Materials, Guangxi Botanical Garden of Medicinal Plants, Nanning, China; ^4^Faculty of Agriculture, Forestry and Food Engineering, Yibin University, Yibin, China; ^5^Genetics Department, Faculty of Agriculture, Zagazig University, Zagazig, Egypt; ^6^Department of Metabolism, Digestion and Reproduction, Imperial College London, London, United Kingdom

**Keywords:** *Centella asiatica*, transcriptome, metabolome, triterpenoid saponin, candidate gene

## Abstract

*Centella asiatica* (L.) Urban is a well-known medicinal plant which has multiple pharmacological properties. Notably, the leaves of *C. asiatica* contain large amounts of triterpenoid saponins. However, there have only been a few studies systematically elucidating the metabolic dynamics and transcriptional differences regarding triterpenoid saponin biosynthesis during the leaf development stages of *C. asiatica.* Here, we performed a comprehensive analysis of the metabolome and transcriptome to reveal the dynamic patterns of triterpenoid saponin accumulation and identified the key candidate genes associated with their biosynthesis in *C. asiatica* leaves. In this study, we found that the key precursors in the synthesis of terpenoids, including DMAPP, IPP and β-amyrin, as well as 22 triterpenes and eight triterpenoid saponins were considered as differentially accumulated metabolites. The concentrations of DMAPP, IPP and β-amyrin showed significant increases during the entire stage of leaf development. The levels of 12 triterpenes decreased only during the later stages of leaf development, but five triterpenoid saponins rapidly accumulated at the early stages, and later decreased to a constant level. Furthermore, 48 genes involved in the MVA, MEP and 2, 3-oxidosqualene biosynthetic pathways were selected following gene annotation. Then, 17 CYP450s and 26 UGTs, which are respectively responsible for backbone modifications, were used for phylogenetic-tree construction and time-specific expression analysis. From these data, by integrating metabolomics and transcriptomics analyses, we identified *CaHDR1* and *CaIDI2* as the candidate genes associated with DMAPP and IPP synthesis, respectively, and *CaβAS1* as the one regulating β-amyrin synthesis. Two genes from the CYP716 family were confirmed as *CaCYP716A83* and *CaCYP716C11*. We also selected two UGT73 families as candidate genes, associated with glycosylation of the terpenoid backbone at C-3 in *C. asiatica*. These findings will pave the way for further research on the molecular mechanisms associated with triterpenoid saponin biosynthesis in *C. asiatica*.

## Introduction

*Centella asiatica* (L.) Urban, a stoloniferous perennial herb of the Umbelliferae family found in tropical and sub-tropical areas (Nguyen et al., 2019), is a well-known medicinal plant used in China, India and Southeast Asia (Bhavna and Jyoti, 2011; Weng et al., 2012). It has been registered in different Pharmacopoeia of the world such as China, India and Europe (Brinkhaus et al., 2000; Kalita et al., 2015). Experimental and clinical investigations show that *C. asiatica* is useful in the treatment of varicose veins, eczema, wound healing disturbances, intellectual disability, ulcer, lupus and striae gravidarum (Liu et al., 2008; Prakash et al., 2017). This is attributed to an enormous amount of triterpenoid saponins, mainly asiaticoside, madecassoside, centelloside and sceffoleoside, which are collectively known as centelloids in *C. asiatica* (Matsuda et al., 2001; James and Dubery, 2009). Therefore, the triterpenoid saponins of *C. asiatica* are vital resources for their important and unique medicinal values. Identification of the relevant biosynthetic pathways will thus be helpful for our further understanding and utilization of these triterpenoid saponins. However, most of the previous studies on these compounds have only focused on their properties, bioactivities and pharmacology, and only a few genes involved in the triterpenoid saponin biosynthesis in *C. asiatica* have been identified, such as HMG-CoA reductase (Kalita et al., 2018), farnesyl diphosphate synthase (Kim et al., 2005a), squalene synthase (Kim et al., 2005b), oxidosqualene cyclase (Kim et al., 2005c), CYP450s (Fukushima et al., 2011; Kim et al., 2018) and UGTs (Kim et al., 2017; Han et al., 2022) and so on. Therefore, the biosynthetic pathways involved have yet to be fully characterized and need to be further investigated at the molecular level.

It is reported that there are more than 300 types of triterpenoid carbon skeletons (Xu et al., 2004; Thimmappa et al., 2014), whose chemical structures are formed from triterpene aglycones (sapogenins) and one or more sugar groups connected by glycosidic bonds (Phillips et al., 2006). Further evidences have supported the notion that there are similar routes of the triterpenoid saponin biosynthesis in different plants (Augustin et al., 2011; Cárdenas et al., 2019), and the core of triterpenoid saponin biosynthetic pathway is well-understood (Xu et al., 2022).

The whole triterpenoid saponin pathway involves three major stages: an initial stage, followed by terpenoid backbone construction and the modification stage (Sawai and Saito, 2011; Zhao et al., 2022). In the first stage, isopentenyl diphosphate (IPP) and its isomer dimethylallyl diphosphate (DMAPP), are formed from two independent pathways: IPP biosynthesis by initially condensing three units of acetyl-CoA in the cytosolic mevalonate (MVA) pathway (Huang et al., 2014), and DMAPP biosynthesis starting from the condensation of pyruvate and phosphoglyceraldehyde in the plastidial methylerythritol phosphate (MEP) pathway (Luo et al., 2016). IPP can be partially isomerized into DMAPP by IPP isomerase (IDI) (Wen et al., 2017). When the terpenoid backbone is constructed, farnesyl diphosphate synthase (FPS) condenses and catalyzes IPP and DMAPP into farnesyl pyrophosphate (FPP). Subsequently, two FPP molecules form squalene by the action of squalene synthase (SS). Under the action of squalene epoxidase (SE), epoxidation of squalene at the second and third carbon positions yields 2,3-oxidosqualene, which is a direct precursor to the construction of the triterpenoid backbones (Haralampidis et al., 2002). Next, oxidosqualene cyclases (OSCs) catalyze the cyclitization of 2,3-oxidosqualene into various triterpenoid backbones, which is thus a critical step of the triterpenoid biosynthesis pathway (Aharoni et al., 2006). During the modification stage, the triterpenoid backbones are chemically modified by cytochrome P450 monooxygenases (CYP450s) through a series of hydroxylation/oxidation reactions and then these are changed by UDP-glycosyltransferases (UGTs) through glycosylation reactions, all of which finally result in the formation of various triterpenoid saponins (Seki et al., 2015; Yang et al., 2018).

Transcriptomics is an effective technical means of mining the key regulatory genes associated with the active ingredients of biosynthetic pathways (Kuwahara et al., 2019). In an earlier study, an analysis of transcriptome sequences of leaves of *C. asiatica* by Illumina (Solexa sequencing technology) was conducted, and this contributed to the mining of the targeted genes related to triterpenoid saponin biosynthesis (Sangwan et al., 2013). The development of long-read sequencing technologies and assembly pipelines has allowed the completion of the whole genome assembly of *C. asiatica* at the chromosome-scale (Pootakham et al., 2021). These complete, accurate and contiguous representative genome sequences can provide valuable references to further understand the triterpenoid saponin biosynthesis in *C. asiatica*. Additionally, the metabolomics database can then be used to provide information regarding the accumulation of various secondary metabolites during the biosynthetic pathways of bioactive ingredient formation (Saito and Matsuda, 2010), and it can also provide reference information for gene mining.

The comprehensive analysis of transcriptomics and metabolomics has been widely applied to delineate the relationships between the dynamic changes of secondary metabolites and the differential expression of corresponding genes during the bioactive ingredients biosynthetic pathways in numerous medicinal plants, such as *Panax notoginseng* (Wei et al., 2018), *Sapindus mukorossi* (Xu et al., 2022), *Platycodon grandiflorus* (Chang et al., 2022), *Dendrobium huoshanense* (Yuan et al., 2022) and *Carthamus tinctorius* (Wang et al., 2021). The medicinal properties of *C. asiatica* are largely attributed to the centelloids that present predominantly in the leaves of *C. asiatica* (Sangwan et al., 2013). However, there have been few studies systematically elucidating the metabolic dynamics and transcriptional differences regarding the triterpenoid saponin biosynthesis during the leaf developmental stages of *C. asiatica* based on triterpenoid saponin metabolism and transcriptomics data.

In this study, we identified the key candidate genes and metabolites by analyzing the expression patterns of differentially expressed genes (DEGs) by transcriptomics and the differentially accumulated metabolites (DAMs) via metabolomics in *C. asiatica* leaves at different growth stages. This study will pave the way for future research on the molecular mechanisms associated with triterpenoid saponin biosynthesis in *C. asiatica*.

## Materials and methods

### Plant materials

The plants of *C. asiatica* were artificially cultivated in the greenhouse at the Guangxi Botanical Garden of Medicinal Plants. The plants were cultivated using an illumination intensity of 300 µmol m^-2^ s^-1^, a relative humidity of 60 ± 2% at 26 ± 2°C with a light/dark cycle of 16/8 h. The leaves of *C. asiatica* were collected from four growth stages and representative examples are shown in **Supplementary Figure S1**. The samples were at the 10, 20, 30 and 40 day leaf-ages with three replicates at each stage and were designated as JXC1-1, JXC1-2, JXC1-3, JXC2-1, JXC2-2, JXC2-3, JXC3-1, JXC3-2, JXC1-3, JXC4-1, JXC4-2 and JXC4-3, respectively. The leaf age was determined by attaching a label to each leaf indicating the date when the leaf first emerged. The number of leaves collected at each stage was subjected to their size, and all the samples were immediately frozen by using liquid nitrogen and then stored at −80°C for subsequent extraction of RNA and metabolites.

### Metabolomics determination and analysis

A wide targeted metabolomics analysis was conducted on the samples from each leaf growth stage to explore the dynamic patterns of metabolites during the developmental stages of *C. asiatica* leaves. This analysis was carried out by MetWare Biotechnology Co., Ltd., Wuhan, China.

Initially, the leaf samples were freeze-dried by using a vacuum freeze-dryer (Scientz-100F, Ningbo, China), and then zirconia beads were added and the mixture were grinded in a mixer mill (MM 400, Retsch, Haan, Deutschland) for 1.5 min, followed by 70% methanol extraction. The suspension was centrifuged (at 2,000 rpm for 3 min) (Anpel, Shanghai, China), and then filtered through a 0.22 μm econofilter. The filtrates were analyzed using a UPLC-ESI-MS/MS system (UPLC, SHIMADZU Nexera X2, MS, Applied Biosystems 4500 Q TRAP). An Agilent SB-C18 column (1.8 µm, 2.1 mm×100 mm) (California, USA) was used for separation at 40°C. Mobile phases of solvents A and B were used and these were composed of pure water with 0.1% formic acid and acetonitrile with 0.1% formic acid, respectively. The gradient conditions were as follows: Phase B started with 5%, followed by 95% at 0 ~ 10 min, and maintained at 95% for another 1.0 min, and finally 5% at 11.1 ~ 14 min,. For each sample, the injection volume was 4 μL at a flow rate of 0.35 mL/min. The effluent was collected and assessed after connection to electrospray ionization (ESI)-triple quadrupole-linear ion trap (QTRAP)-MS.

The operating parameters for ESI were as follows: the source temperature was 550°C and the ion spray voltage (IS) was set to 5,500 V (positive ion mode)/-4,500 V (negative ion mode). The ion source gases I and II as well as the curtain gas were set at 50, 60 and 25 psi, respectively, and the collision-activated dissociation was set to high. Ten and 100 μmol/L polypropylene glycol solutions were used to tune the instrument and to perform mass calibration in the triple quadrupole (QQQ) as well as the linear ion trap modes, respectively. Scans of the QQQ were acquired in the form of multiple reaction monitoring (MRM) experiments with nitrogen as the collision gas and this was set to medium. The declustering potential (DP) and collision energy (CE) for each MRM transition were performed after further optimizations. A specific set of MRM transitions was used to monitor each period based on the metabolites found to be eluted within that period.

To assess the metabolite diversity between and within groups, all the metabolic data were subjected to multivariate statistical analysis, including unsupervised principal component (PCA) and orthogonal partial least squares discriminant analyses (OPLS-DA). After evaluation of these results, the metabolites with |Log_2_ (FC)| ≥ 1, *p*-value < 0.05, and variable importance in the project (VIP) ≥ 1 were identified as DAMs.

### RNA extraction, Illumina sequencing and determination of DEGs

RNA-seq was carried out by MetWare Biotechnology Co., Ltd. (Wuhan, China). Total RNA was extracted from the four stages of *C. asiatica* leaves and its quality was assessed by running on 1% agarose gels and using the NanoPhotometer^®^ spectrophotometer (IMPLEN, CA, USA). RNA concentration was measured using the Qubit^®^ RNA Assay Kit in Qubit^®^2.0 Flurometer (Life Technologies, CA, USA). RNA integrity was determined using the RNA Nano 6000 Assay Kit of the Bioanalyzer 2100 system (Agilent Technologies, CA, USA). One μg of RNA from each sample was used for sample preparation. cDNA libraries were generated using the NEBNext^®^UltraTM RNA Library Prep Kit purchased from Illumina^®^ (NEB, USA) by following the manufacturer’s instructions. Raw sequence reads with nucleotides with uncertain base information as well as those with low-quality sequence reads were filtered using FASTP v 0.19.3 to produce clean reads. These clean reads were then mapped to the whole genome of *C. asiatica* (unpublished data) to acquire the genes using HISAT v2.1.0.

The expression levels of the genes were calculated after normalization to the number of fragments per kilobase of transcript per million fragments (FPKM). Genes were further analyzed using functional annotations and pathway analyses to seven public databases: KEGG, KOG, GO, Pfam, Tremble, NR and Swiss-Prot. The levels of DEGs were analyzed using the original count data from each sample which was obtained by their expression quantification with DESeq2 software. Those genes with Benjamini-Hochberg-adjusted *p* value < 0.05 and |log_2_ (FC)| > 1 were identified as DEGs.

### Gene identification and phylogenetic analysis of the CYP450 and UGT families

Two methods were used for gene identification of the CYP450 and UGT families in the *C. asiatica* genome. Firstly, the protein sequences from the chromosome-scale genome of *C. asiatica* (unpublished data) were used to construct a local protein database. The known protein sequences of *Arabidopsis thaliana* for 242 CYP450s and 117 UGTs were collected from the Arabidopsis Information Resource (TAIR, https://www.arabidopsis.org/). These were used to search the local protein database of *C. asiatica* by using BLASTP, which is a basic local environment alignment search tool for proteins. The cut-off E-value used was 10^-10^. Subsequently, the Hidden-Markov Model (HMM) files for P450.hmm (PF00067) and UGT.hmm (PF00201) which were downloaded from the Pfam database (https://pfam.xfam.org/), were used to search the local protein database of *C. asiatica* to find all predicted CYP450 and UGT family members via the HMMER 3.0 (https://www.hmmer.org/) program with a cut-off E-value of 10^-10^. After integrating the results of the above two methods, the remaining sequences were assumed as the candidate CYP450 and UGT family members of *C. asiatica*, after removing the redundant sequences. The candidate family members were further verified via PfamScan (e-value = 0.001, https://www.ebi.ac.uk/Tools/pfa/pfamscan/).

Phylogenetic analysis was performed to align the full-length protein sequences of CYP450 and UGT from *Arabidopsis* and CYP450 and UGT from *C. asiatica* by using the MEGA software (version 7.0) with default parameters, respectively. A phylogenetic tree was constructed by using the MEGA software with the 1,000-replicate neighbor-joining (NJ) method and the results were illustrated by using the interactive tree of life online software (https://itol.embl.de/help.cgi).

### Integration of transcriptome and metabolome data by WGCNA and Pearson correlation analysis

To better outline relationships of the data between the genes and related metabolites involved in triterpenoid saponin biosynthesis, weighted gene coexpression network analysis (WGCNA) and Pearson correlation analysis were conducted with the default parameters in the free online data analysis, Metware Cloud Platform (https://cloud.metware.cn). The soft threshold, the network heatmap plot of genes, the module heatmap and the trait-related modules were constructed by using WGCNA. The correlation results obtained from Pearson correlation analysis were mapped in a correlation cluster heatmap.

### Real-time quantitative PCR analysis

Eighteen DEGs associated with the triterpenoid saponin biosynthesis were selected for real-time quantitative PCR (RT-qPCR) analysis relative to the reference gene, *CaGAPDH*. cDNA was synthesized using the SuperScript^®^ III Reverse Transcriptase kit by following the manufacturer’s protocols. RT-qPCR was performed on a Bio-Rad CFX96 RT-qPCR platform (Bio-Rad Laboratories, Hercules, CA, USA) using SYBR^®^ Premix Ex Taq™ kit (Takara, Dalian, China) according to the manufacturer’s protocols. The relative expression levels of DEGs were calculated using the 2^-ΔΔCT^ method and then normalized against *CaGAPDH*. The primers used are listed in **Supplementary Table S1**.

## Results

### Metabolic profiles of *C. asiatica* leaves at four different growth stages

In order to evaluate the metabolite variations in the leaves at different growth stages, a wide targeted metabolomics analysis was performed using samples collected at four time-points during leaf development. A clustering heatmap analysis was performed from the whole metabolite database, which showed that the three replicates used in this study had a high similarity (**Supplementary Figure S2A**). PCA analysis also showed a distinct separation in leaf samples from the four different stages, indicating that these metabolites presented a dynamic change pattern with the leaf growth (**Supplementary Figure S2B**). These results indicated that the metabolite detection methods used in this study were reliable.

**Figure 1:**
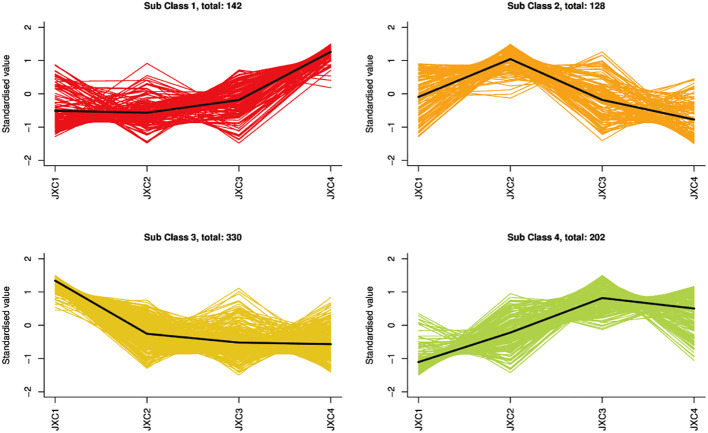
The k-means analysis of metabolites.

A total of 802 metabolites were obtained, mainly containing terpenoids (68), flavonoids (84), alkaloids (44), amino acids and their derivatives (96), phenolic acids (135), nucleotides and their derivatives (49), lignans (18), coumarins (26), tannins (1), organic acids (68) and lipids (108) (**Supplementary Table S2**). To further understand the complexity of the different metabolites at each leaf growth stage, these metabolites were divided into four clusters by using a k-mean clustering method. Terpenoids were mainly found in clusters 3 and 2, with the highest accumulation of metabolites belonging to these clusters being from JXC1 and JXC2, respectively (**Figure 1**).

The 263 metabolites were mapped to the reference pathways according to KEGG annotations, and these were annotated to 96 pathways into three categories. A total of 91 pathways were associated with metabolism, and three and two pathways were involved with environmental information and genetic information processing, respectively. For the “metabolism” term, the top priority was “global and overview maps” (228 metabolites), followed by “amino acid metabolism” (89 metabolites), “carbohydrate metabolism” (69 metabolites) and “biosynthesis of other secondary metabolites” (67 metabolites) (**Supplementary Figure S3A**).

### Analysis of the metabolites involved in the biosynthesis of triterpenoid saponins in the *C. asiatica* leaves at four different growth stages

The key precursors in the biosynthesis of terpenoids, including DMAPP (Wmjp001948), IPP (Wmjn000963) and β-amyrin (Wmjn007463), were detected and they showed differential accumulation in the *C. asiatica* leaves at the four different growth stages (**Supplementary Table S3**). Gradual increased trends were observed in their contents from JXC1 to JXC4 with a peak at JXC4 (**Supplementary Table S3**).

In the downstream metabolites of triterpenoid saponin biosynthesis, a total of 32 triterpenes and 14 triterpenoid saponins were tentatively detected (**Supplementary Table S3**). Hierarchical cluster analysis (HCA) based on the relative levels of triterpenes (**Supplementary Figure S4A**) and triterpenoid saponins (**Supplementary Figure S4B**) in the *C. asiatica* leaves at the four growth stages showed that the levels of most of these compounds were markedly changed during leaf development, and this further confirmed the PCA results. Additionally, a total of 22 triterpenes were found to be differentially accumulated (**Supplementary Table S4**), which were divided into 7 groups based on their accumulation levels. The 12 triterpenes in cluster A had their levels significantly decreased from stage JXC3, and three of these compounds in cluster B showed high accumulations at stage JXC3 (**Supplementary Figure S5A**). A total of eight triterpenoid saponins showed differential accumulation (**Supplementary Table S4**), which were divided into four groups. In cluster A, the five triterpenoid saponins showed high accumulation at stage JXC1 and then tapered off from stages JXC2 to JXC4. For cluster B, the accumulation of one triterpenoid saponin showed a downtrend trend, with the lowest level seen at stage JXC3, but then it increased by stage JXC4. Cluster C was similar to cluster B, where lower levels were seen at stages JXC2 and JXC3. The content of triterpenoid saponins in cluster D appeared to increase continuously during leaf growth (**Supplementary Figure S5B**).

### Transcriptome profiles of *C. asiatica* leaves at four different growth stages

In this study, in order to explore gene expression patterns during the four different stages of leaf development, and to identify genes participating in the biosynthetic pathways of triterpenoid saponins at the transcriptional level, 12 cDNA libraries, in batches of three, were prepared and analyzed. The 12 leaf samples generated 56.59 million high-quality clean reads which constituted 84.89 GB of cDNA sequence data. The Q20 and Q30 values ranged from 98.09 to 98.46% and 94.03 to 95.00%, respectively, and the GC content ranged from 43.50 to 44.92%. The reads in each library were aligned to the *C. asiatica* reference genome with an average match ratio of 82.71% (**Table 1**).

**Table 1:** Summary statistics of the RNA-seq results.

Sample	Raw Reads	Clean Reads	Clean Base(G)	Q20(%)	Q30(%)	GC Content(%)	Mapped Reads	Unique mapped
JXC1-1	44495124	43049490	6.46	98.13	94.18	44.07	85.65%	83.70%
JXC1-2	50987344	49197288	7.38	98.09	94.03	43.99	84.86%	82.99%
JXC1-3	45120822	44072352	6.61	98.17	94.24	44.02	84.39%	82.42%
JXC2-1	54430828	51992362	7.8	98.34	94.75	44.18	84.83%	82.25%
JXC2-2	48504278	46719306	7.01	98.13	94.14	43.73	78.94%	76.89%
JXC2-3	49317248	47190664	7.08	98.29	94.55	44.02	80.63%	78.35%
JXC3-1	49342226	47165158	7.07	98.46	95	43.84	82.78%	80.64%
JXC3-2	53689036	51481462	7.72	98.35	94.67	43.75	83.03%	80.71%
JXC3-3	50530798	49049278	7.36	98.32	94.61	43.92	83.96%	81.60%
JXC4-1	48555886	46378168	6.96	98.34	94.65	43.63	81.49%	79.45%
JXC4-2	46447748	44272732	6.64	98.34	94.77	43.69	83.52%	81.45%
JXC4-3	47132200	45318910	6.8	98.31	94.52	43.5	78.42%	76.61%

### Identification of DEGs

A total of 22,594 genes expressed were obtained in at least one sample with FPKM > 0 (**Supplementary Table S5**). The PCA results showed most of the samples at the same stages were highly correlated, indicating that the gene expression patterns were also highly reproducible (**Supplementary Figure S6**). Six comparative groups were constructed to demonstrate the differentiation at the transcriptional level at different growth stages of *C. asiatica* leaves: JXC1_vs_JXC2, JXC1_vs_JXC3, JXC1_vs_JXC4, JXC2_vs_JXC3, JXC2_vs_JXC4 and JXC3_vs_JXC4, and a total of 3889 DEGs were identified (**Supplementary Table S6**). Among these DEGs, the numbers of up- and down-regulated DEGs for each comparison group are shown in **Table 2**. There were more down-regulated DEGs than up-regulated ones in all the compared groups except for JXC3_vs_JXC4. Compared with JXC4, JXC3 contained a smaller number of DEGs, with 253 and 238 up- and down-regulated DEGs, respectively. The highest number of DEGs were identified in the JXC1_vs_JXC4 comparison, which showed 945 and 1,960 up- and down-regulated DEGs, respectively. In summary, there were significant differences in the number of DEGs presented in the six comparative groups.

**Table 2:** The counts of differentially expressed genes.

Group	Total	Down	Up
JXC1_vs_JXC2	1800	1236	564
JXC1_vs_JXC3	2281	1555	726
JXC1_vs_JXC4	2905	1960	945
JXC2_vs_JXC3	498	338	160
JXC2_vs_JXC4	1372	829	543
JXC3_vs_JXC4	491	238	253

### GO enrichment and KEGG pathway analysis

The functions of DEGs were annotated by GO enrichment analysis, which were enriched within three main ontologies: biological process (BP), cellular component (CC) and molecular function (MF). Finally, the 3,389 DEGs of all samples were annotated to 3,623 GO terms (**Supplementary Figure S7**). Among them, the BP category contained 2,151 GO terms (59.37%), of which the major terms were the cellular (GO: 0009987), metabolic (GO: 0008152), single-organism (GO: 0044699) and biological processes (GO: 0050789)). In addition, some of these DEGs also responded to stimuli (GO: 0050896), biological regulation (GO: 0065007) and localization (GO: 0051179). The CC category contained 387 GO terms, including the membrane (GO: 0016020 and 0044425), cell (GO: 0005623 and 0044464) and organelle (GO: 0043226). The MF category contained 1,085 GO terms (29.95%), including mainly catalytic (GO: 0003824), binding (GO: 0005488), transporter (GO: 0005215) and nucleic acid binding transcription factor activities (GO: 0001071).

Moreover, the DEGs were also mapped to the KEGG pathways by using the KEGG database. The 3,389 DEGs in all samples were annotated to 126 KEGG pathways into five categories. There were three, four, 18, two and 99 pathways for cellular processes, environmental information processing, genetic information processing, organismal systems and metabolism, respectively (**Supplementary Figure S3B**). The main enriched pathways associated with secondary metabolism in each comparison group in our study were: biosynthesis of secondary metabolites (ko01110), metabolic pathways (ko01100), sesquiterpenoid and triterpenoid biosynthesis (ko00940), terpenoid backbone biosynthesis (ko00900), phenylpropanoid biosynthesis (ko00940) and flavonoid biosynthesis (ko00941). These enriched pathways provided valuable information for mining candidate genes associated with triterpenoid saponin biosynthesis.

### DEGs expression patterns associated with triterpenoid backbone biosynthesis

**Figure 2:**
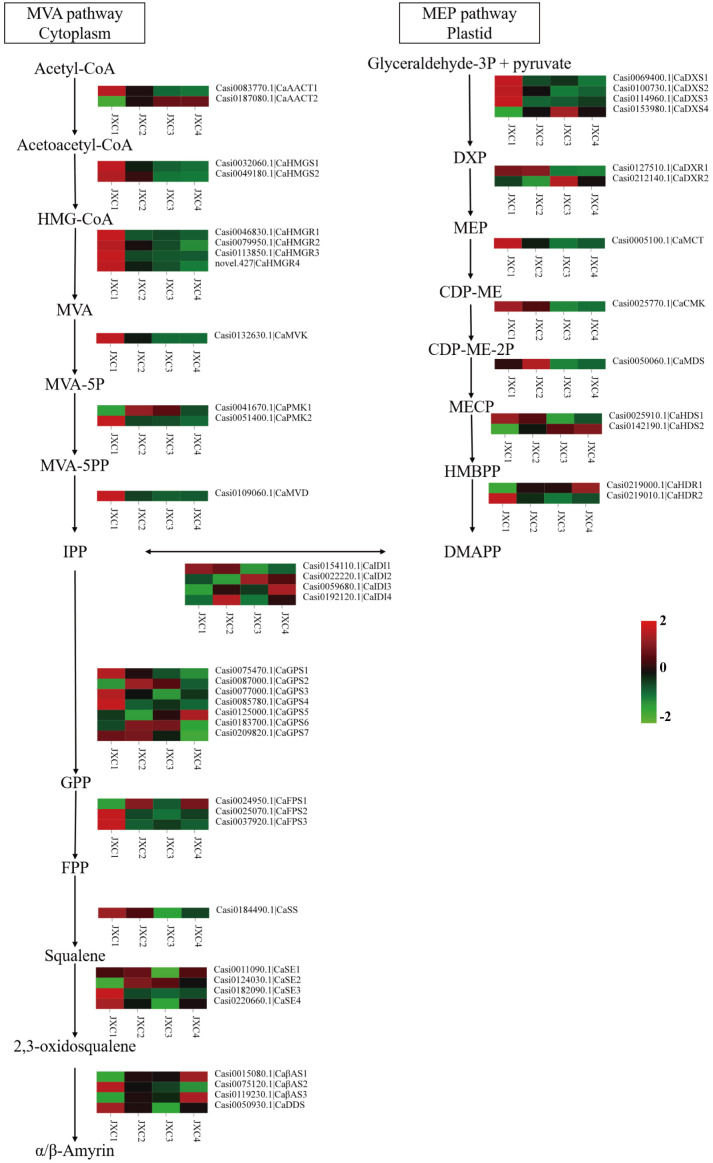
The proposed pathways for triterpenoid saponin biosynthesis in *C. asiatica*. The expression levels of the gene encoding enzymes that catalyze each step of the triterpenoid saponin biosynthesis pathway are shown.

To further explore the molecular mechanism of triterpene and triterpenoid saponin biosynthesis in the *C. asiatica* leaves, the expression levels of genes associated with their biosynthesis were analyzed. From the transcriptomics analysis, we found 48 genes were associated with the terpenoid backbone biosynthetic pathways, all of which could be classified into the MVA, MEP and 2, 3-oxidosqualene pathways (**Figure 2**).

The KEGG pathway analysis showed that a total of 12 genes encoding six enzymes were associated with the MVA pathway: two acetyl-CoA C-acetyltransferase (AACTs), two 3-hydroxy-3-methylglutaryl-CoA (HMG-CoA) synthetases (HMGSs), four HMG-CoA reductases (HMGRs), one mevalonate kinase (MVK), two phosphomevalonate kinases (PMKs) and one metholvalic-5-diphosphate decarboxylase (MVD). The MEP pathway, starting with pyruvate and glyceraldehyde3-phosphate, consisted of 17 genes encoding eight enzymes: four 1-deoxy-D-xylulose-5-phosphate (DXP) synthetases (DXSs), two DXP reductoisomerases (DXRs), one 4-cytidine diphosphate-2-C-methyl-D-erythritol (CDP-ME) synthetase (MCT), 1 CDP-ME kinase (CMK), one 2-C-methyl-D-erythritol-2,4-cyclic phosphate synthetase (MDS), two (E)-4-hydroxy-3-methyl-2-butenyl-pyrophosphate (HMBPP) synthetases (HDSs), two HMBPP reductases (HDRs) and four isopentenyl pyrophosphate (IPP) isomerases (IDIs). The expression levels were highest in JXC1 for most of these genes, except for *CaAACT2*, *CaMVK* and *CaPMK1* which were involved in the MVP pathway and for *CaDXS4*, *CaDXR2*, *CaMDS*, *CaHDS2*, *CaHDR1* and 3 *CaIDIs* involved in the MEP pathway.

Additionally, 19 genes encoding six enzymes were involved in carbocyclic biosynthesis: seven geranyl pyrophosphate (GPP) synthetases (GPSs), three farnesyl pyrophosphate(FPP) synthetase (FPSs), one squalene synthase (SS), four squalene epoxidases (SEs), three β-amyrin synthases (βASs) and one dammarenediol-II synthase (DDS). However, these genes had variable expression levels at the different developmental stages.

### Identification and phylogenetic analysis of CYP450s and UGTs associated with backbone modifications

Through our genome-wide analysis, we identified 231 CYP450s in the *C. asiatica*. By using the CYP450 reference database, they were classified into 9 clades, 41 families, with 48.05 and 51.95% being the A- and non-A-types of CYP450s, respectively (**Supplementary Table S7**). For further analysis of their functional roles and evolutionary relationships in *C. asiatica*, 231 CYP450s along with 242 CYP450s from *A. thaliana* were employed in generating phylogenetic trees for the A- (**Supplementary Figure 8A**) and non-A-types (**Supplementary Figure 8B**) CYP450s, respectively. All the A-types of CYP450s were represented by the CYP71 clans. These consisted of 111 genes belonging to 18 families, which were CYP71, 73, 75-79, 81-P84, 89, 93, 98, 701, 703, 706 and 712. The members of non-A-type CYP450s were divided into eight clans according to the phylogenetic tree, including five single-family clans (CYP51, 74, 710, 97 and 711) and three multifamily clans (CYP72, 85 and 86). CYP72, 85 and 86 were the largest three clans, containing 48, 37 and 27 CYP450s, respectively.

By using genome-wide analysis, we identified 140 UGTs in the *C. asiatica*. We aligned the *C. asiatica* UGT amino acid sequences with the 117 UGTs obtained from *Arabidopsis* and was able to construct a phylogenetic tree (**Supplementary Table S8**). We ultimately classified these UGTs into 15 groups and 20 families, among which three with amino acid sequences less than 40% identities to any representative sequences could not be classified. The UGT85 family of Group G was the largest family with 19 genes, and the next largest group was the UGT73 family of Group D with 18 genes (**Supplementary Figure 9**).

**Figure 3:**
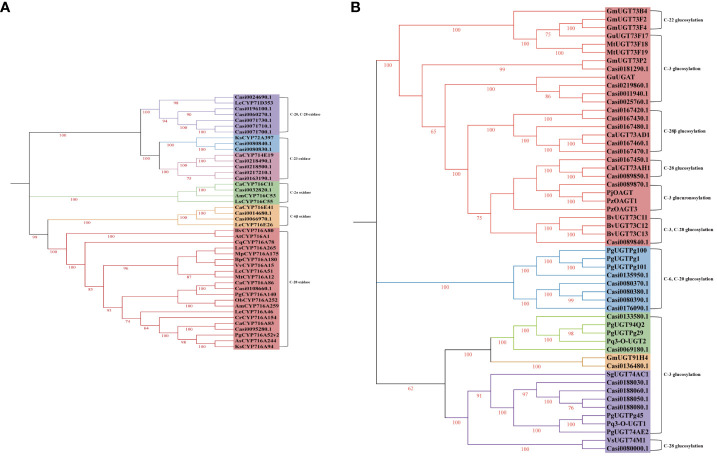
The phylogenetic tree of previously characterized triterpenoid biosynthesis CYP450s **(A)**, UGTs **(B)** and those CYP450s **(A)** and UGTs **(B)** isolated from *C. asiatica* in this study.

It has been reported that the members of the CYP85, CYP72 and CYP71 families are primary CYP450s which are associated with triterpenoid saponin biosynthesis in dicotyledonous plants. We identified five *CYP716s* of CYP85 family genes (one *CYP716C* gene, two *CYP716E* genes and two *CYP716A* genes), six CYP72 family genes (two *CYP72A* genes and four *CYP714E* genes) and six *CYP71D* genes by using the transcriptomic data obtained from *C. asiatica*. According to the results of the phylogenetic analysis (**Figure 3A**), Casi0095280.1, Casi0032820.1 and Casi0108660.1 had 100.00, 100.00 and 87.62% identities to CaCYP716A83, CaCYP716C11 and CaCYP716A86, respectively. Casi0014680.1 and Casi0066970.1 exhibited 100.00 and 69.58% sequence identities with CaCYP716E41, respectively. Casi0218490.1, Casi0218500.1, Casi0163190.1 and Casi0217210.1 exhibited 94.79, 79.23, 42.27 and 40.32% sequence identities with CaCYP714E19, respectively. Casi0080830.1 and Casi0080840.1 exhibited 64.63 and 63.85% sequence identities with KsCYP72A397, respectively.

In recent studies, several UGT family members (UGT71, UGT73, UGT74, UGT91 and UGT94) were shown to be associated with triterpenoid saponin biosynthesis. Here, we screened five UGT71, 13 UGT73, five UGT74, one UGT91 and two UGT94 family gene members via the transcriptomic data obtained from *C. asiatica*. According to the results of the phylogenetic analysis (**Figure 3B**), Casi0167460.1, Casi0167470.1, Casi0167480.1, Casi0167430.1 and Casi0167420.1 exhibited 99.60, 85.11, 72.95, 52.92 and 47.29% sequence identities with CaUGT73AD1, respectively. Casi0089850.1 and Casi0167450.1 exhibited 100.00 and 64.88% sequence identities with CaUGT73AH1, respectively.

### Construction of correlated gene co-expression networks by using WGCNA

**Figure 4:**
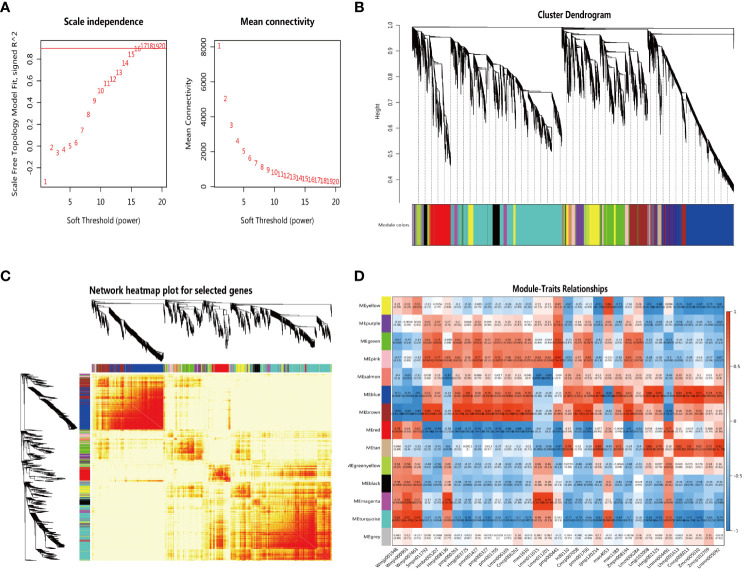
The network topology analysis for different soft threshold powers **(A)** The gene clustering dendrogram with adjacency-based dissimilarity, together with assigned module colors **(B)** The construction of module heatmap **(C)** The construction of the relationships between modules and traits **(D)**.

To identify genes related to triterpenoid saponin biosynthesis, low-expressed genes (FPKM value ≤ 0) were filtered out, and a total of 15,808 genes obtained were used for the construction of gene co-expression networks by WGCNA. Our results showed that a soft threshold power of 16 was the lowest power with a scale-free topology model fit index of 0.90 and relatively high mean connectivity (**Figure 4A**). Gene clustering dendrogram was constructed and detected 13 gene co-expression modules labeled with different colors. Genes in the same module indicated that their expression patterns were similar (**Figures 4B, C**).

To determine the correlation relationship between gene co-expression modules and 33 metabolites involved in the triterpenoid saponin biosynthesis, the heatmap of module-traits relationships was constructed (**Figure 4D**). We observed that the ‘turquoise’ module had the highest correlation with three key precursors in the biosynthesis of terpenoids, DMAPP (Wmjp001948) (r = 0.82, p = 0.0011), IPP (Wmjn000963) (r = 0.91, p < 0.001) and β-amyrin (Wmjn007463) (r = 0.95, p < 0.001). The ‘brown’ module was associated with 18 triterpenes and had the highest correlation with 13 triterpenes (r > 0.83, p < 0.001). The ‘blue’ module was associated with 7 triterpenoid saponins, of which 5 had the highest correlation (r > 0.84, p < 0.001).

### Pearson correlation analysis between the concentrations of triterpenoid saponins and expressions of their biosynthesis-related genes

**Figure 5:**
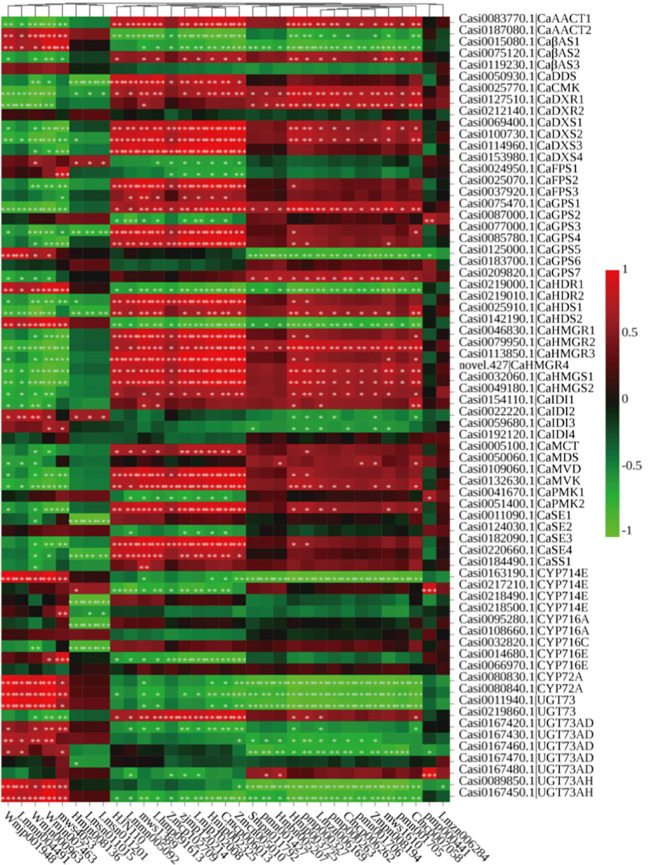
The Pearson correlation analysis between genes and metabolites in the triterpenoid saponin pathway. Significant ones p < 0.05 marked with *, p < 0.01 marked with **, p < 0.001 marked with ***.

The expression levels of the 68 candidate genes and the metabolite concentrations involved in triterpenoid saponin biosynthesis were analyzed by Pearson correlation (**Figure 5**). The results indicated that DMAPP (Wmjp001948), IPP (Wmjn000963) and β-amyrin (Wmjn007463), the key precursors in the biosynthesis of terpenoids, were significantly and positively correlated with *HDR1* (Casi0219000.1) (r = 0.78, p = 0.0026), *IDI2* (Casi0022220.1) (r = 0.79, p = 0.0023) and *CaβAS1* (Casi0015080.1) (r = 0.91, p < 0.001). Our results showed that Casi0095280.1 and Casi0108660.1 had 100.00 and 87.62% identities to CaCYP716A83 and CaCYP716A86, respectively, and these CYPs catalyzed α-amyrin/β-amyrin to ursolic acid/oleanolic acid. Ursolic acid (mws4053) was positively correlated with Casi0095280.1. In addition, corosolic (mws1610) and maslinic acids (Zmpn008194) showed positive correlations with Casi0032820.1, which had 100.00% identity to CaCYP716C11. Finally, we found that saikosaponin L (Zmcp102709) and medicagenic acid-3-O-glucosyl-(1,6)-glucosyl-(1,3)-glucoside (Lmmn004491) were significantly and positively correlated with Casi0219860.1 (r = 0.85, p = 0.0004) and Casi0011940.1 (r = 0.93, p < 0.001), respectively, both of which were members of UGT73 family and had high degrees of identity with GuUGA.

### Verification of DEGs related to triterpenoid saponins by qRT-PCR measurements

**Figure 6:**
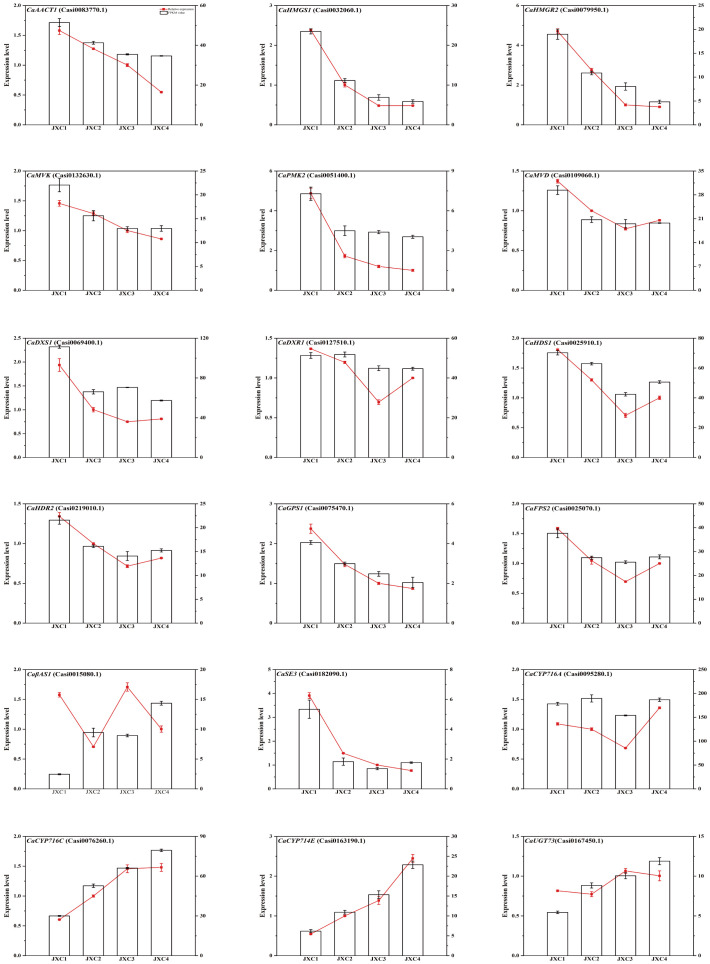
The qRT-PCR analysis of the potential genes involved in triterpenoid saponin biosynthesis.

To further verify the transcriptomics data obtained, the relative expression levels of 18 genes associated with triterpene and triterpenoid saponin biosynthesis in *C. asiatica* were selected for analysis by RT-qPCR. These included six genes (*CaAACT1*, *CaHMGS1*, *CaHMGR2*, *CaMVK*, *CaPMK2* and *CaMVD*) involved in the MVP pathway, four genes (*CaDXS1*, *CaDXR1*, *CaHDS1* and *CaHDR2*) involved in the MEP pathway, four genes (*CaGPS1*, *CaFPS2*, *CaSE3* and *CaβAS1*) involved in the terpenoid backbone construction stage and four genes (*CaCYP714E*, *CaCYP716A*, *CaCYP716C* and *CaUGT73*) involved in terpenoid backbone modification. The results showed that the majority of these genes exhibited consistent expression patterns with the transcriptomics data (**Figure 6**), which supported the analysis we performed.

## Discussion

The triterpenes and triterpenoid saponins of *C. asiatica* have been regarded as its essential bioactive compounds due to their important medicinal values in the treatment of various chronic disorders (Prakash et al., 2017; Puttarak et al., 2017). However, previous studies regarding these compounds from *C. asiatica* have only focused on their chemical structures, bioactivity assessments and cultivation (Prasad et al., 2019). Relatively, few studies regarding the accumulation of metabolites as well as the molecular mechanisms of their biosynthesis in *C. asiatica* have been conducted. Therefore, it is important to assess the biosynthetic pathways of triterpenes and triterpenoid saponins in *C. asiatica* in order to achieve the optimal production of these bioactive compounds from the plants. The majority of the triterpenes and triterpenoid saponins are known to be predominantly accumulated in the leaves of *C. asiatica* (Sangwan et al., 2013). Here, we identified the metabolites and genes of *C. asiatica* leaves at different growth stages based on transcriptomics and metabolomics data, in order to gain novel insights into the accumulation and biosynthesis of triterpenes and triterpenoid saponins.

Structurally, the triterpenes and triterpenoid saponins in the *C. asiatica* belong to the terpenoid group, and they have a similar precursor synthesis pathway to those of the other terpenoids. These are thought to be derived through a cross-talk between the MVA and MEP pathways (Patel et al., 2016; Li et al., 2022). We identified 12 genes in the MVA pathway and these involved six key enzymes and 17 genes in the MEP pathway as well as the involvement of eight other key enzymes from our transcriptomics analysis. These genes were further analyzed in combination with the metabolomics analysis, and the expression levels of *CaHDR1* (Casi0219000.1) and *CaIDI2* (Casi0022220.1) were found to be consistent with the concentrations of DMAPP (Wmjn000963) and IPP (Wmjp001948), respectively.

HDR, the limiting enzyme in the MEP pathway, catalyzes HMBPP into a mixture of 5:1 IPP and DMAPP (Adam et al., 2002). Overexpression of the *HDR* genes in *Arabidopsis* has been shown to increase the production of IPP and DMAPP (Botella-Pavía et al., 2010). Both IPP and DMAPP are the essential precursors at regulatory branching points in the biosynthesis of terpenoids (Sun et al., 2010), but the relatively unreactive IPP can be interconverted to its highly electrophilic isomer, DMAPP, by the catalysis of IDI. This compound provides both the electrophilic primer substrate and the condensation substrate for terpenoid biosynthesis (Chen et al., 2012). Therefore, the function of IDI is considered to be the critical first step that controls the overall biosynthesis of all terpenoids (Ramos-Valdivia et al., 1997; Zhu et al., 2014). Previous studies reported that overexpression of the *IDI* gene generated an accumulation of several related terpenoids by acting downstream in the MEP pathway (Kajiwara et al., 1997; Sun et al., 1998). All these reports suggested that IDI may be a target enzyme for regulating terpenoid biosynthesis (Chen et al., 2012). Our results also showed that the up-regulation of *CaHDR1* and *CaIDI2* promoted the accumulation of IPP and DMAPP, suggesting that these genes play pivotal roles in the regulation of the biosynthesis of these compounds in *C. asiatica*.

The OSCs are considered to play key roles in the formation of the different triterpenoid skeletons (Basyuni et al., 2007), because they are the key initial steps for catalyzing 2,3-oxidosqualene cyclization into diverse forms of the triterpenoid skeletons (Mao et al., 2023). This is supported by experiments whereby the enzyme activities were altered and differential effects on triterpene synthesis were observed (Morita et al., 2000). The down-regulation of the expression of the *β-AS* gene in *Panax ginseng* hairy roots has also been shown to reduce β-amyrin and oleanane-type ginsenoside (Zhao et al., 2015). In addition, α-AS from *Pisum sativum*, a multifunctional triterpene synthase, generated α- and β-amyrins by heterologous expression in yeast (Iturbe-Ormaetxe et al., 2003). Similarly, in our study, the expression level of *CaβAS1* (Casi0015080.1) was consistent with the concentration of β-amyrin (Wmjn007463). In addition, the sequence of *CaβAS1* had a high identity (79.79%) with the known *C. asiatica βAS*, which indicated that *CaβAS1* may catalyze 2,3-oxidosqualene to β-amyrin in *C. asiatica.*

The triterpenoid skeletons result from carboxyl modification by the CYP450s and then the saccharide side chains are normally introduced by the UGTs leading to the biosynthesis of different triterpene saponins (Xu et al., 2022). The members of CYP450s associated with triterpenoid saponin biosynthesis are mainly the CYP85, CYP72 and CYP71 families. For instance, CYP716s of the CYP85 family catalyze the successive three-step oxidation of α-amyrin, lupel and β-amyrin at C-28 (Shang and Huang, 2019). CYP72s (CYP72A and CYP714E) catalyze the hydroxylation of oleanolic acid/ursolic acid at C-23 (Han et al., 2018) and the members of CYP71D subfamily are involved in the successive three-step oxidation of 20-hydroxylupeol in *Lotus japonicus* to produce 20-hydroxybetulinic acid (Krokida et al., 2013). It has been shown that CYP716As catalyze the successive three-step oxidation of the α-amyrin/β-amyrin/lupeol backbone to form hydroxyl, aldehyde and carboxyl moieties at C-28 (Xu et al., 2022). In this study, two CYP716As, Casi0095280.1 and Casi0108660.1, showed 100.00 and 87.62% identities to CaCYP716A83 and CaCYP716A86, respectively, and these can catalyze α-amyrin/β-amyrin to ursolic acid/oleanolic acid (Miettinen et al., 2017). Our results also showed that ursolic acid (mws4053) was more abundant at the 20 and 40 day stages of leaf development, which was similar to the expression pattern of Casi0095280.1. In addition, Casi0032820.1 had 100.00% identity to CaCYP716C11, which is known to catalyze the hydroxylation of ursolic acid/oleanolic acid/6β-hydroxy-oleanolic acid at C-2α to corosolic acid/maslinic acid/6β-hydroxy-maslinic acid (Miettinen et al., 2017). In our study, corosolic (mws1610) and maslinic acids (Zmpn008194) from our metabolomics data also showed they accumulated at the 10 and 20-day stages of leaf development, which was consistent with the up-regulation of Casi0032820.1 at JXC1 and JXC2.

It was reported that CaCYP716E41 can catalyze the hydroxylation of ursolic acid/oleanolic acid/maslinic acid at C-6β to 6β-hydroxy-ursolic acid/6β-hydroxy-oleanolic acid/6β-hydroxy-maslinic acid in *C. asiatica* (Miettinen et al., 2017). Although the products of CaCYP716E41 were not found in our metabolomics data, Casi0014680.1 and Casi0066970.1 exhibited 100.00% and 69.58% sequence identities with CaCYP716E41, respectively, according to our results of the phylogenetic analysis. Therefore, Casi0066970.1 may also be a candidate gene involved in triterpene biosynthesis in *C. asiatica*.

We also found that four candidate CYP72As (Casi0218490.1, Casi0218500.1, Casi0163190.1 and Casi0217210.1) exhibited 94.79, 79.23, 42.27 and 40.32% sequence identities with CaCYP714E19, respectively. In addtion, Casi0080830.1 and Casi0080840.1 exhibited 64.63 and 63.85% sequence identities with KsCYP72A397, respectively, and it catalyzes the hydroxylation of ursolic acid/oleanolic acid at C-23. This suggested that these CYP72As may catalyze ursolic acid/oleanolic acid to hederagenin/23-hydroxy ursolic acid in *C. asiatica* (Han et al., 2018; Liu et al., 2019). We also found an accumulation of hederagenin (Hmbn005207). However, the change in expression pattern of this compound was significantly and negatively correlated with Casi0163190.1, Casi0080830.1 and Casi0080830.1. A previous study had shown similar results, where it was found that CqCYP716A15 levels were negatively correlated with the triterpene content of *Chenopodium quinoa* (Zhao et al., 2022). These data inferred that CYP72As had multiple regulatory roles during the biosynthesis of triterpenes in *C. asiatica.*

The UGT73 clan is the best candidate family for triterpenoid saponin biosynthesis. We identified five genes (*Casi0167460.1*, *Casi0167470.1*, *Casi0167480.1*, *Casi0167430.1* and *Casi0167420.1*) belonging to the UGT73 clan which showed high degrees of identity with CaUGT73AD1. This enzyme transfers glucuronic acid at C-28β of asiatic acid/madecassic acid to form 28β-Glc-asiatic acid/28β-Glc-madecassic acid (De Costa et al., 2017), suggesting that they catalyze the glucuronosylation of the C28β-hydroxyl group for the biosynthesis of triterpenoid saponins in *C. asiatica*. In addition, the other two members of the UGT73 clan (Casi0089850.1 and Casi0167450.1) exhibited 100.00 and 64.88% sequence identities with CaUGT73AH1, respectively. This enzyme is known to transfer glucuronic acid at C-28 of asiatic acid/UDP-Glc to form 28-Glc-asiatic acid (Kim et al., 2017). This indicates that these genes may catalyze the glucuronosylation of the C28-hydroxyl group for the biosynthesis of triterpenoid saponins in *C. asiatica*. However, the expression levels of the above seven UGT73 candidates were not significantly different during the different stages of leaf development. We found 10 triterpenoid saponins glycosylated at the C-28 position from metabolomics data, most of which also showed no significant difference during the different developmental stages. Therefore, these candidate UGT73 genes and their key intermediates involved in the triterpenoid saponin biosynthetic pathway of *C. asiatica* need to be studied further.

Our study also found two candidate UGT73 DEGs (*Casi0219860.1* and *Casi0011940.1*) which catalyzed glucuronic acid at the C-3 position and showed high degrees of identity to GuUGA (Xu et al., 2016). The expression pattern of *Casi0219860.1*, which showed a high expression level and accumulation at the 10 day-stage of leaf development, was consistent with the level found in saikosaponin L (Zmcp102709). In addition, the expression pattern of *Casi0011940.1*, which showed high expression levels and accumulation at the 30 and 40 day-stages of leaf development, was consistent with the level of medicagenic acid-3-O-glucosyl-(1,6)-glucosyl-(1,3)-glucoside (Lmmn004491). This result infers that Casi0219860.1 and Casi0011940.1 are most likely to be involved in the biosynthesis of triterpenoid saponins that are glycosylated at the C-3 position in *C. asiatica*.

## Conclusions

Despite the multiple pharmacological properties associated with triterpenes and triterpenoid saponins found in *C. asiatica* leaves, relatively few systematic studies have been undertaken to elucidate the biosynthesis of these compounds during their leaf developmental stages. In our investigation, we used the transcriptome and metabolome data to perform a comprehensive analysis to reveal the dynamic patterns of triterpenoid saponin accumulation and the identity of the key candidate genes associated with their biosynthesis in *C. asiatica* leaves. We found that the decrease in levels of the majority of triterpenes occurred only at the late stages of leaf development. However, the majority of triterpenoid saponins rapidly accumulated at early stages of leaf development, and then decreased but remained constant during the later periods. The levels of triterpenes and triterpenoid saponins showed different dynamic patterns at different leaf growth stages, indicating that the whole triterpenoid saponin biosynthetic pathway is relatively complex. Furthermore, 48 genes involved in the synthesis of the terpenoid backbone as well as 17 CYP450s and 26 UGTs genes were found to be associated with the backbone modifications of these compounds. By integrating metabolomics and transcriptomics analyses, we identified five candidate genes as having crucial roles in the biosynthesis of triterpenoid saponins in *C. asiatica*. The relative expression levels of these key genes were further verified by qRT-PCR. Overall, these results will provide a valuable reference for further research on the molecular mechanisms associated with triterpenoid saponin biosynthesis in *C. asiatica* as well as other medicinal plants.

## Data availability statement

The datasets presented in this study can be found in online repositories. The names of the repositories and accession number can be found at the Genome Sequence Archive (GSA), accession number: CRA012378 by using following link: https://ngdc.cncb.ac.cn/search/?dbId=gsa&q=CRA012378.

## Author contributions

LW: Conceptualization, Data curation, Funding acquisition, Investigation, Writing – original draft. QH: Writing – original draft. CL: Investigation, Writing – review & editing, Funding acquisition. HY: Investigation, Writing – review & editing. GT: Data curation, Funding acquisition, Investigation, Writing – review & editing. SW: Data curation, Writing – review & editing. AE-S: Writing – review & editing. SS: Writing – review & editing. KZ: Investigation, Writing – review & editing. LP: Investigation, Writing – review & editing. ZZ: Data curation, Funding acquisition, Writing – review & editing. ML: Conceptualization, Writing – review & editing.

## Supplementary material

Supplementary Figure 1 The *C. asiatica* leaves at four different growth stages.

Supplementary Figure 2 A correlation heatmap of all the samples **(A)**; The metabolomics sample replicates resulting from PCA analysis **(B)**.

Supplementary Figure 3 The KEGG pathway annotation of the metabolites detected **(A)**; The KEGG pathway annotations of the differentially expressed genes **(B)**.

Supplementary Figure 4 The hierarchical clustering for the dynamic change pattern of triterpenes **(A)** and triterpenoid saponins **(B)** in the *C. asiatica* at four leaf growth stages.

Supplementary Figure 5 Upper: temporal expression patterns of the differentially accumulated triterpenes, Lower: a heatmap of triterpenes accumulation levels in different clusters **(A)**; Upper: temporal expression patterns of the differentially accumulated triterpenoid saponins, Lower: a heatmap of the triterpenoid saponins accumulation levels in different clusters **(B)**.

Supplementary Figure 6 The transcriptomic sample replicates resulting from PCA analysis.

Supplementary Figure 7 The GO classification of the differentially expressed genes.

Supplementary Figure 8 The phylogenetic tree of A-type **(A)** and non-A-type **(B)** CYP450s from *C. asiatica* and Arabidopsis. The representative CYP450 family members from *C. asiatica* and Arabidopsis are marked with red circles and blue triangles, respectively.

Supplementary Figure 9 The phylogenetic tree of UGTs from *C. asiatica* and Arabidopsis. The representative UGT family members from *C. asiatica* and Arabidopsis are marked with red circles and blue triangles, respectively.
