# Correction: Integrated metabolome and transcriptome analysis identifies candidate genes involved in triterpenoid saponin biosynthesis in leaves of *Centella asiatica* (L.) Urban

**DOI:** 10.3389/fpls.2026.1784161

**Published:** 2026-02-03

**Authors:** Lingyun Wan, Qiulan Huang, Cui Li, Haixia Yu, Guiyu Tan, Shugen Wei, Ahmed H. El-Sappah, Suren Sooranna, Kun Zhang, Limei Pan, Zhanjiang Zhang, Ming Lei

**Affiliations:** 1 Guangxi Key Laboratory for High-Quality Formation and Utilization of Dao-Di Herbs, Guangxi Botanical Garden of Medicinal Plants, Nanning, China; 2 National Center for Traditional Chinese Medicine (TCM) Inheritance and Innovation, Guangxi Botanical Garden of Medicinal Plants, Nanning, China; 3 National Engineering Research Center for the Development of Southwestern Endangered Medicinal Materials, Guangxi Botanical Garden of Medicinal Plants, Nanning, China; 4 Faculty of Agriculture, Forestry and Food Engineering, Yibin University, Yibin, China; 5 Genetics Department, Faculty of Agriculture, Zagazig University, Zagazig, Egypt; 6 Department of Metabolism, Digestion and Reproduction, Imperial College London, London, United Kingdom

**Keywords:** *Centella asiatica*, transcriptome, metabolome, triterpenoid saponin, candidate gene

The funder “the Guangxi Science and Technology Base and Talent Project”, “Guike AD22080016” to “Ming Lei” was erroneously omitted.

The correct **Funding** statement is “The author(s) declare financial support was received for the research, authorship, and/or publication of this article. This research was funded by the Guangxi Science and Technology Base and Talent Project (Guike AD22080016), the Natural Science Foundation of Guangxi Province (2023GXNSFAA026330 and 2020GXNSFAA159151), Guangxi Science and Technology Base and Special Talents (Guike AD22035026), Scientific Research Funding Project of Guangxi Botanical Garden of Medicinal Plants (GYJ202008), Innovative Team for Traditional Chinese Medicinal Materials Quality of Guangxi (GZKJ2305) and the Key Laboratory Construction Program of Guangxi Health commission (ZJC2020003).”

The original version of this article has been updated.

