# Supplementary figures and images for: Integrated metabolome and transcriptome analysis identifies candidate genes involved in triterpenoid saponin biosynthesis in leaves of Centella asiatica (L.) Urban
Source: Front Plant Sci. 2024 Jan 12;14:1295186. doi: 10.3389/fpls.2023.1295186 (PMC10811118; doi:10.3389/fpls.2023.1295186)

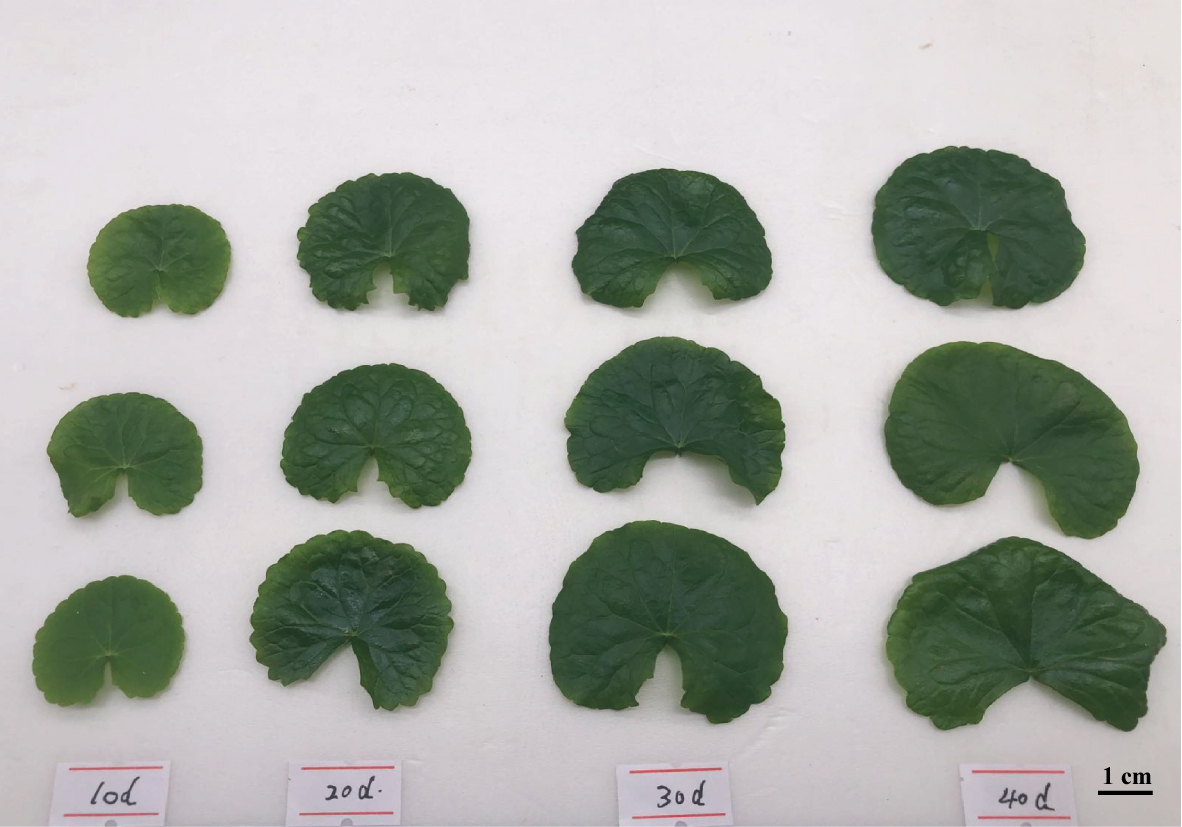

Supplement: Supplementary Figure 1 — The C. asiatica leaves at four different growth stages. [file Image_1.jpeg]

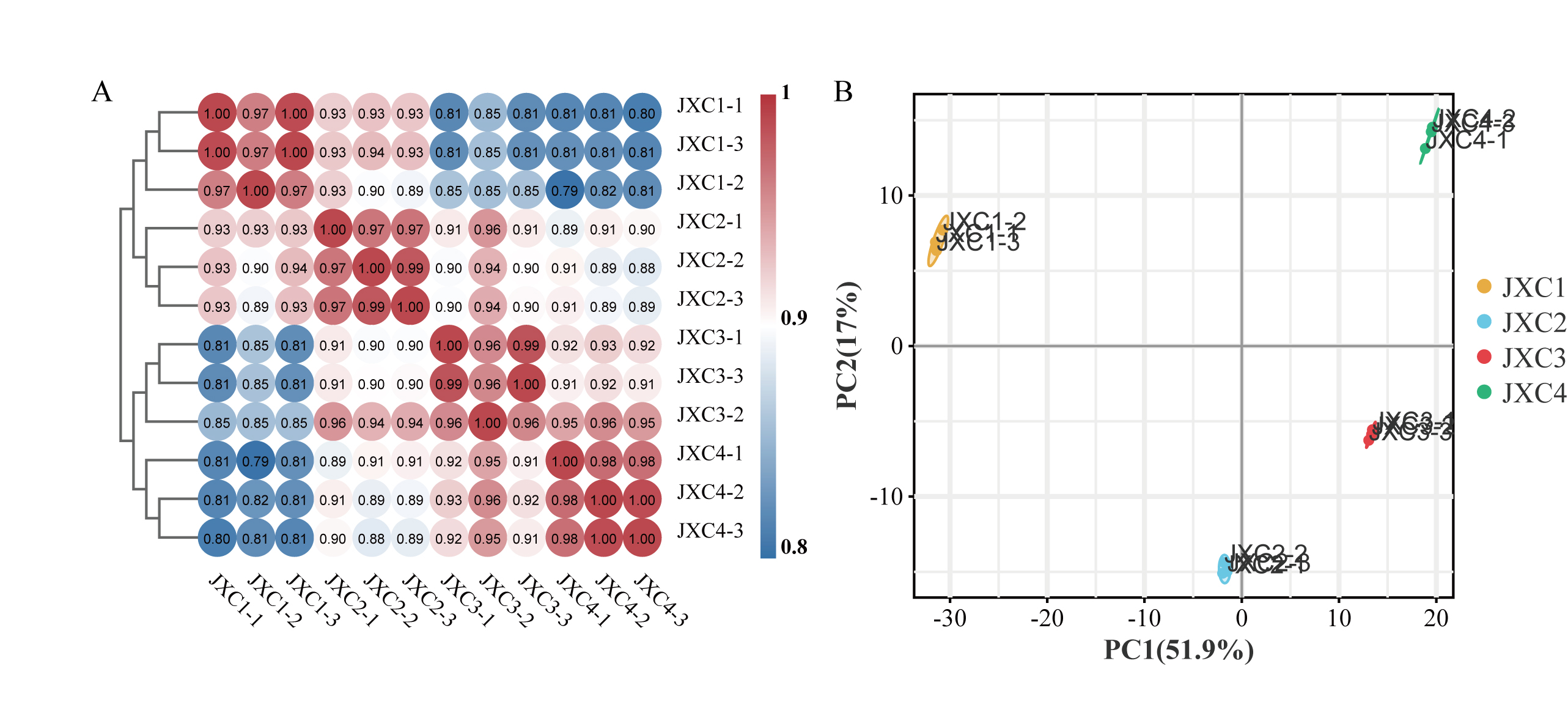

Supplement: Supplementary Figure 2 — A correlation heatmap of all the samples (A); The metabolomics sample replicates resulting from PCA analysis (B). [file Image_2.jpeg]

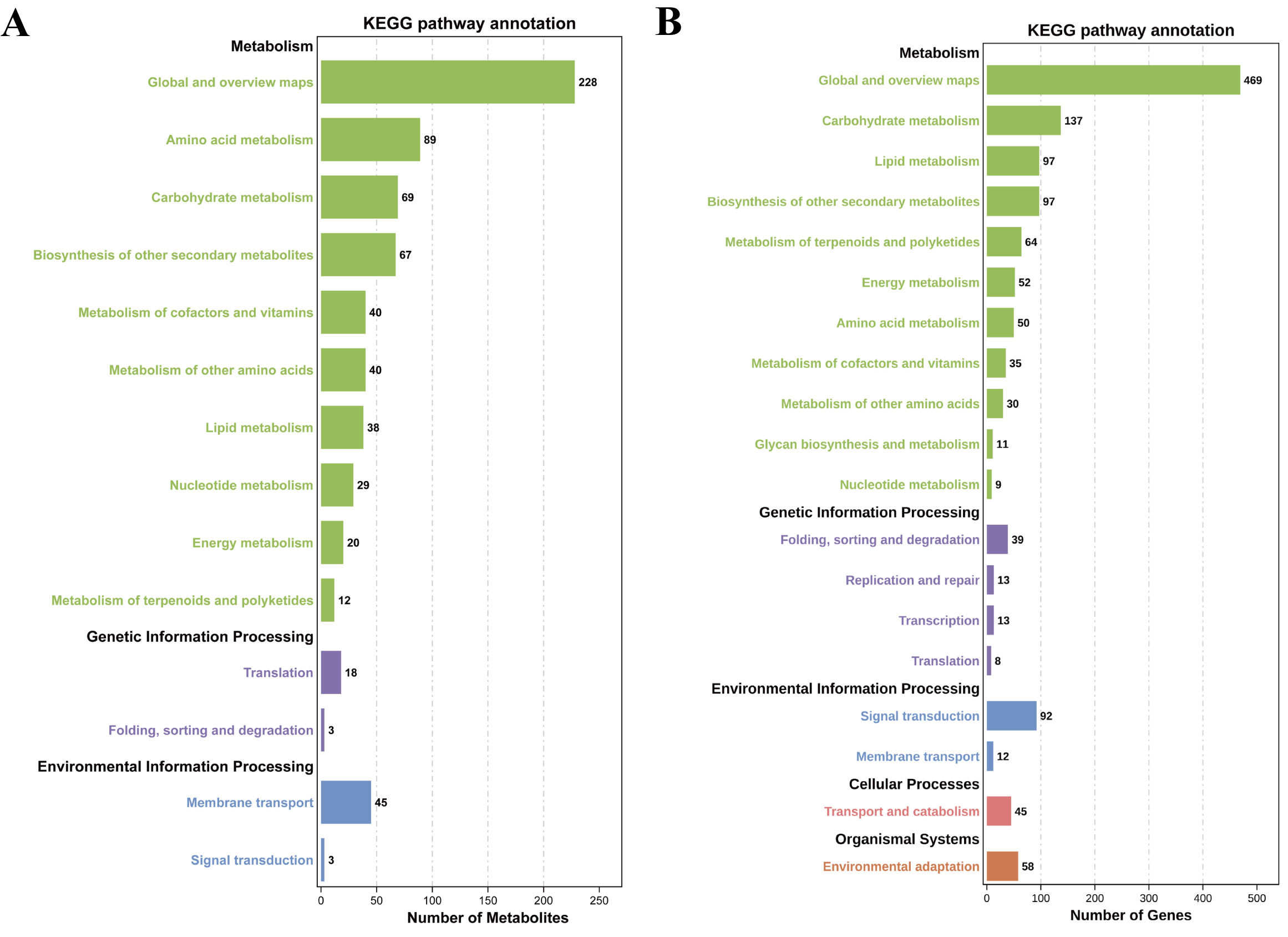

Supplement: Supplementary Figure 3 — The KEGG pathway annotation of the metabolites detected (A); The KEGG pathway annotations of the differentially expressed genes (B). [file Image_3.jpeg]

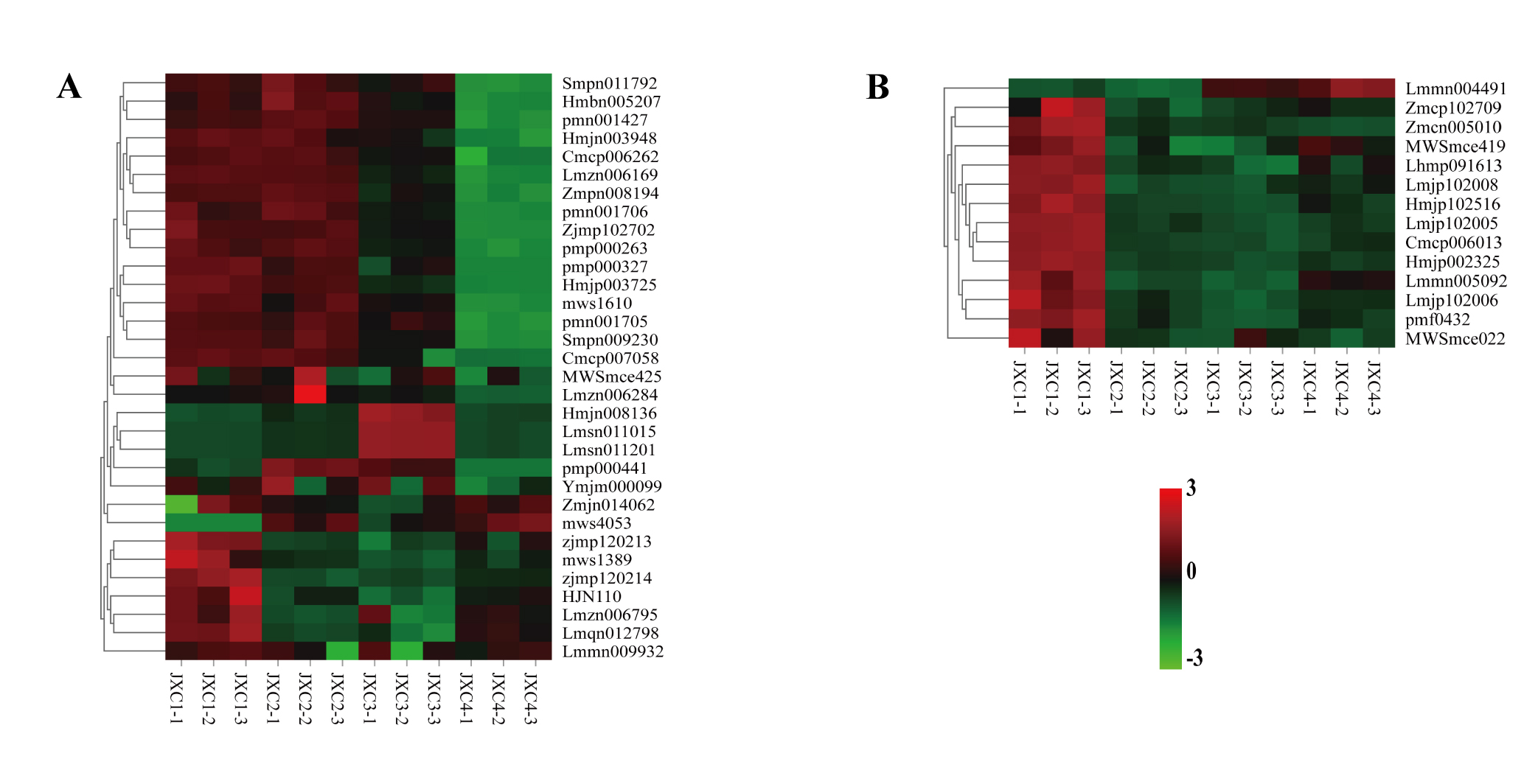

Supplement: Supplementary Figure 4 — The hierarchical clustering for the dynamic change pattern of triterpenes (A) and triterpenoid saponins (B) in the C. asiatica at four leaf growth stages. [file Image_4.jpeg]

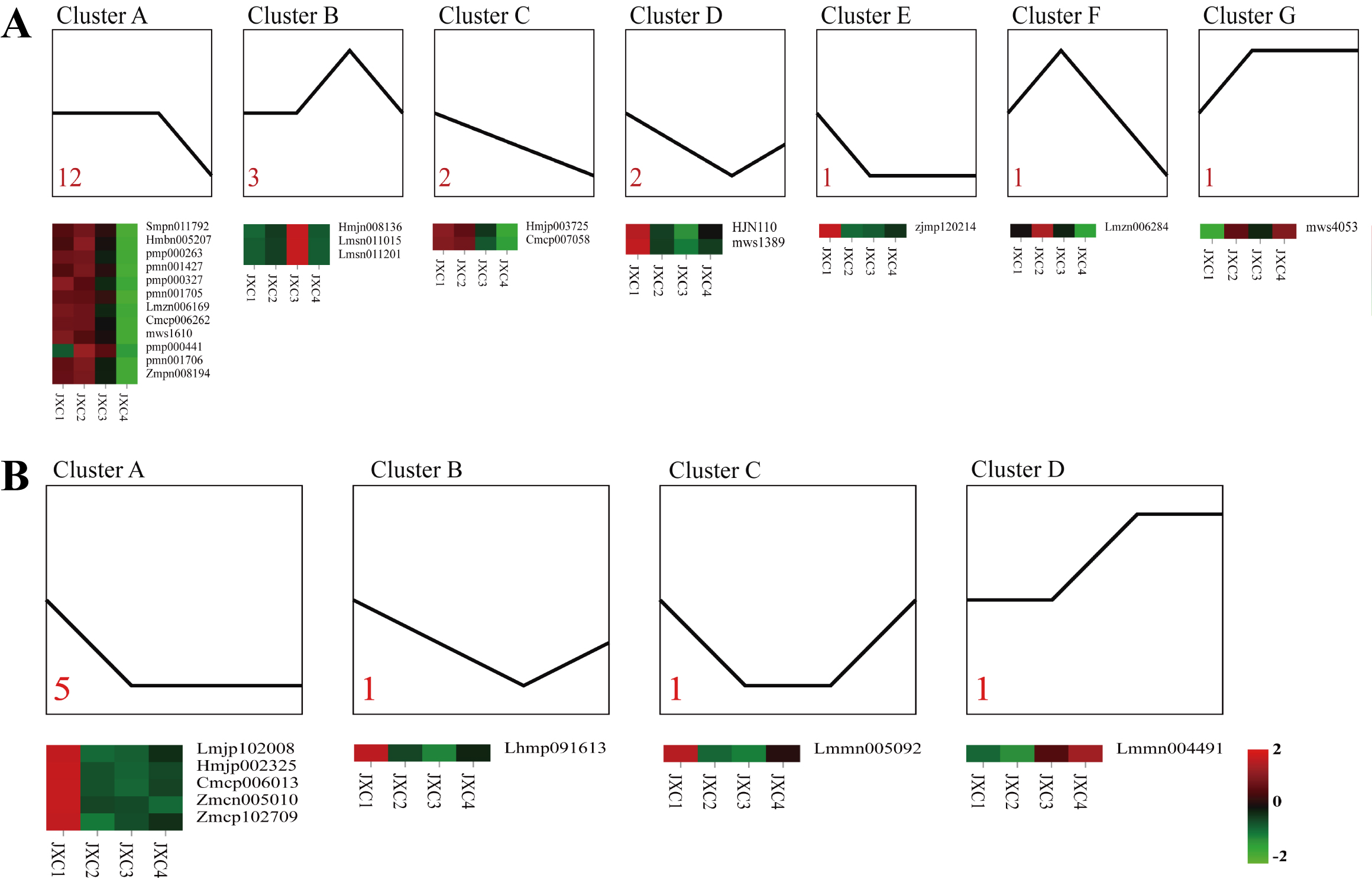

Supplement: Supplementary Figure 5 — Upper: temporal expression patterns of the differentially accumulated triterpenes, Lower: a heatmap of triterpenes accumulation levels in different clusters (A); Upper: temporal expression patterns of the differentially accumulated triterpenoid saponins, Lower: a heatmap of the triterpenoid saponins accumulation levels in different clusters (B). [file Image_5.jpeg]

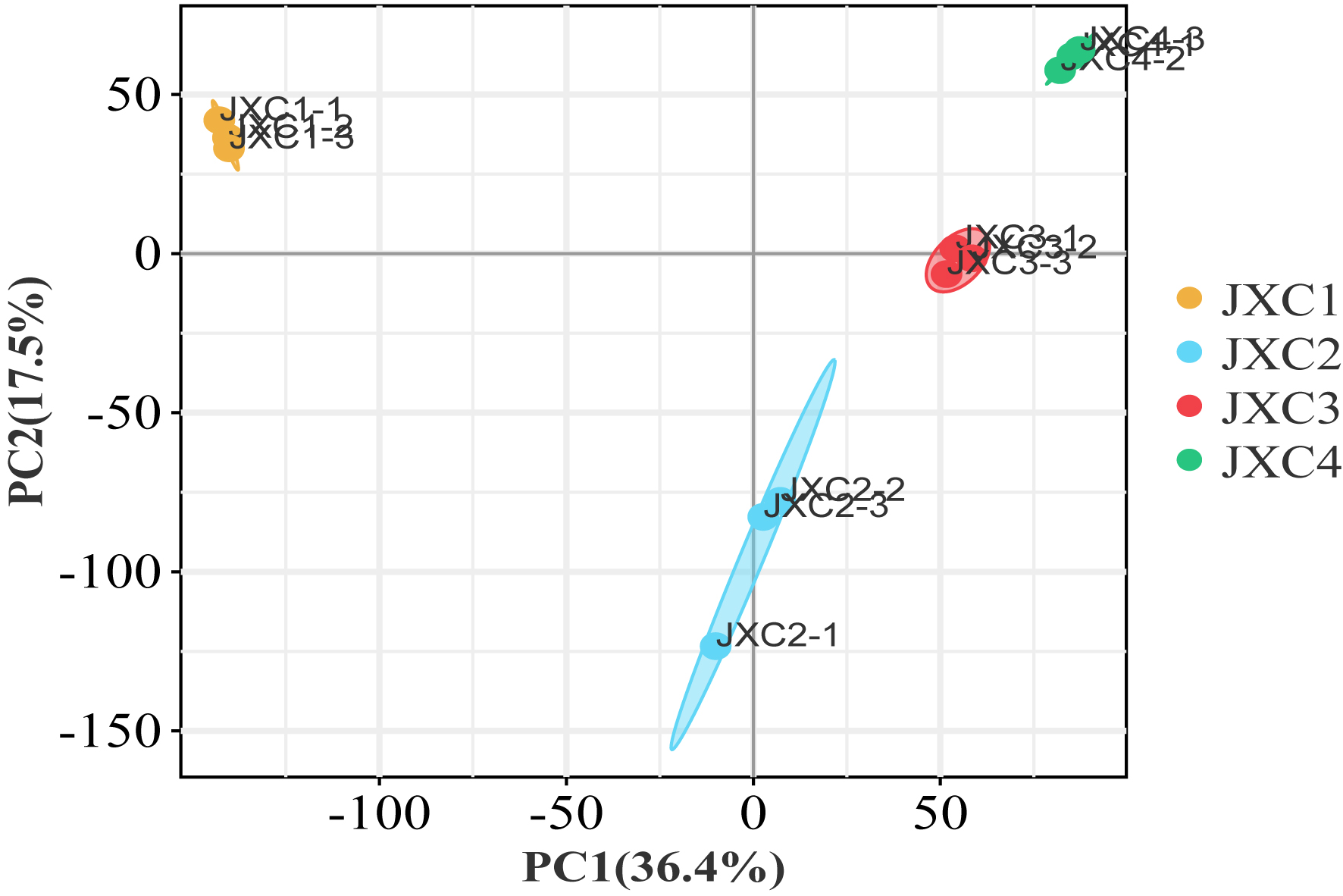

Supplement: Supplementary Figure 6 — The transcriptomic sample replicates resulting from PCA analysis. [file Image_6.jpeg]

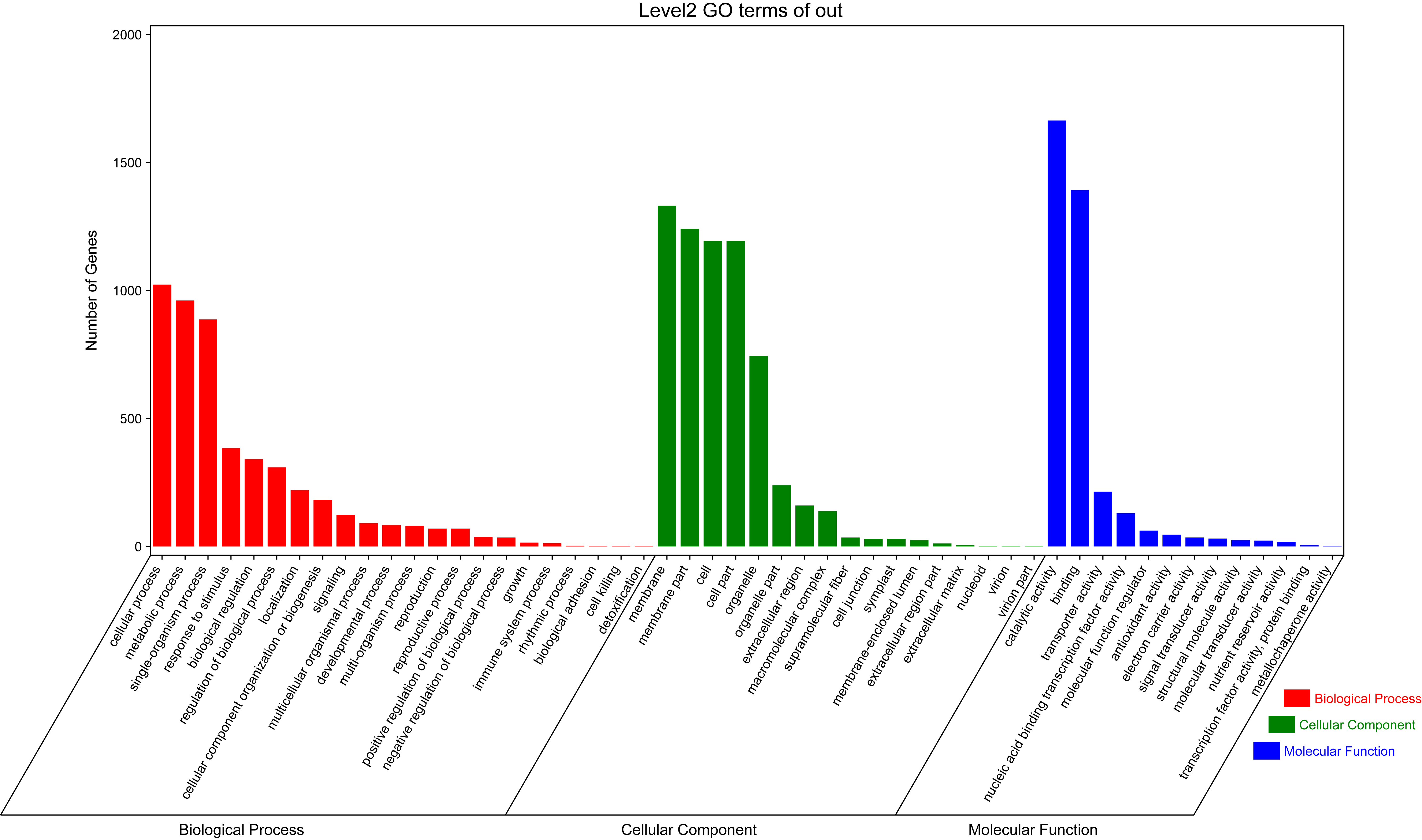

Supplement: Supplementary Figure 7 — The GO classification of the differentially expressed genes. [file Image_7.jpeg]

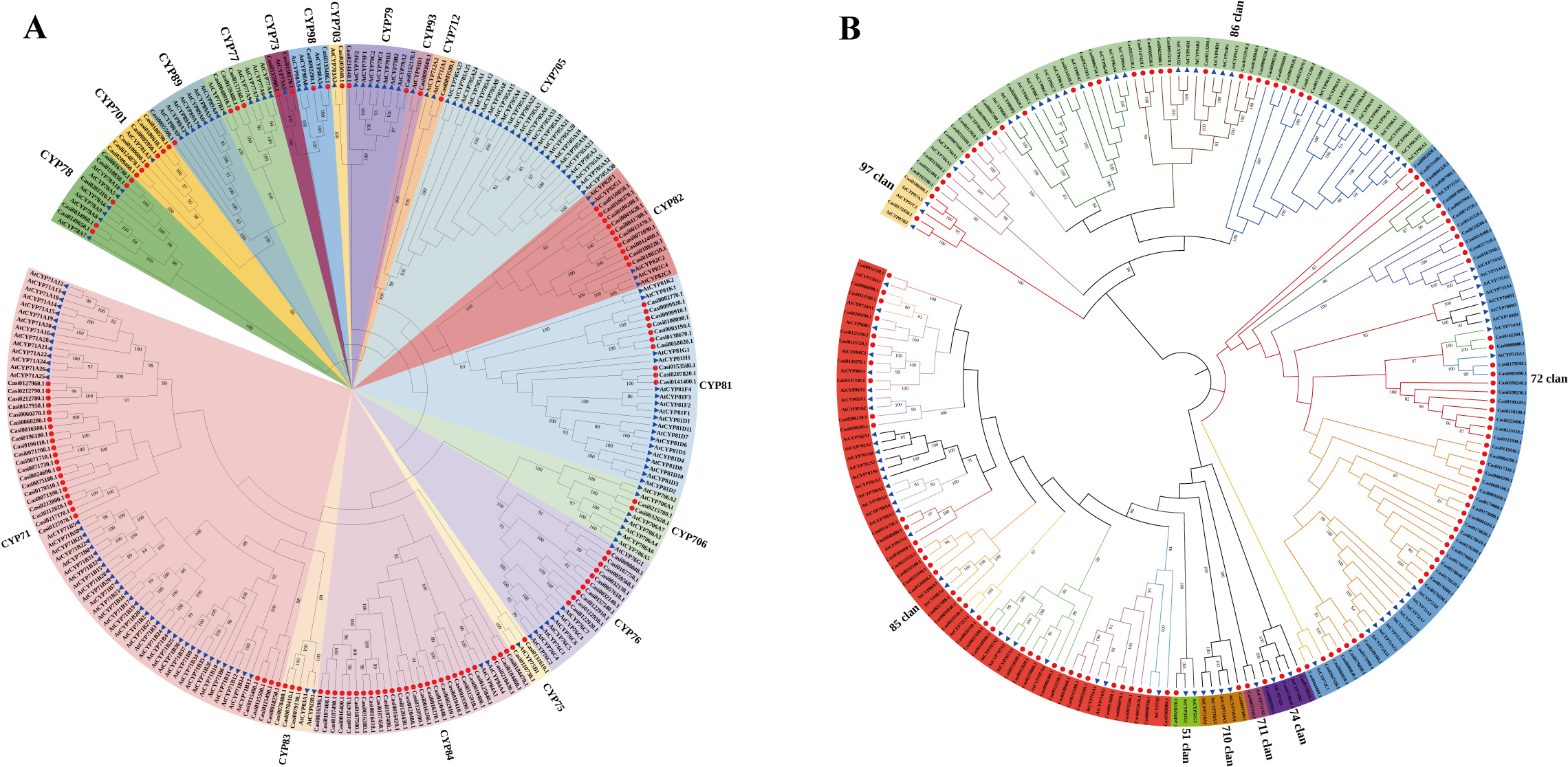

Supplement: Supplementary Figure 8 — The phylogenetic tree of A-type (A) and non-A-type (B) CYP450s from C. asiatica and Arabidopsis. The representative CYP450 family members from C. asiatica and Arabidopsis are marked with red circles and blue triangles, respectively. [file Image_8.jpeg]

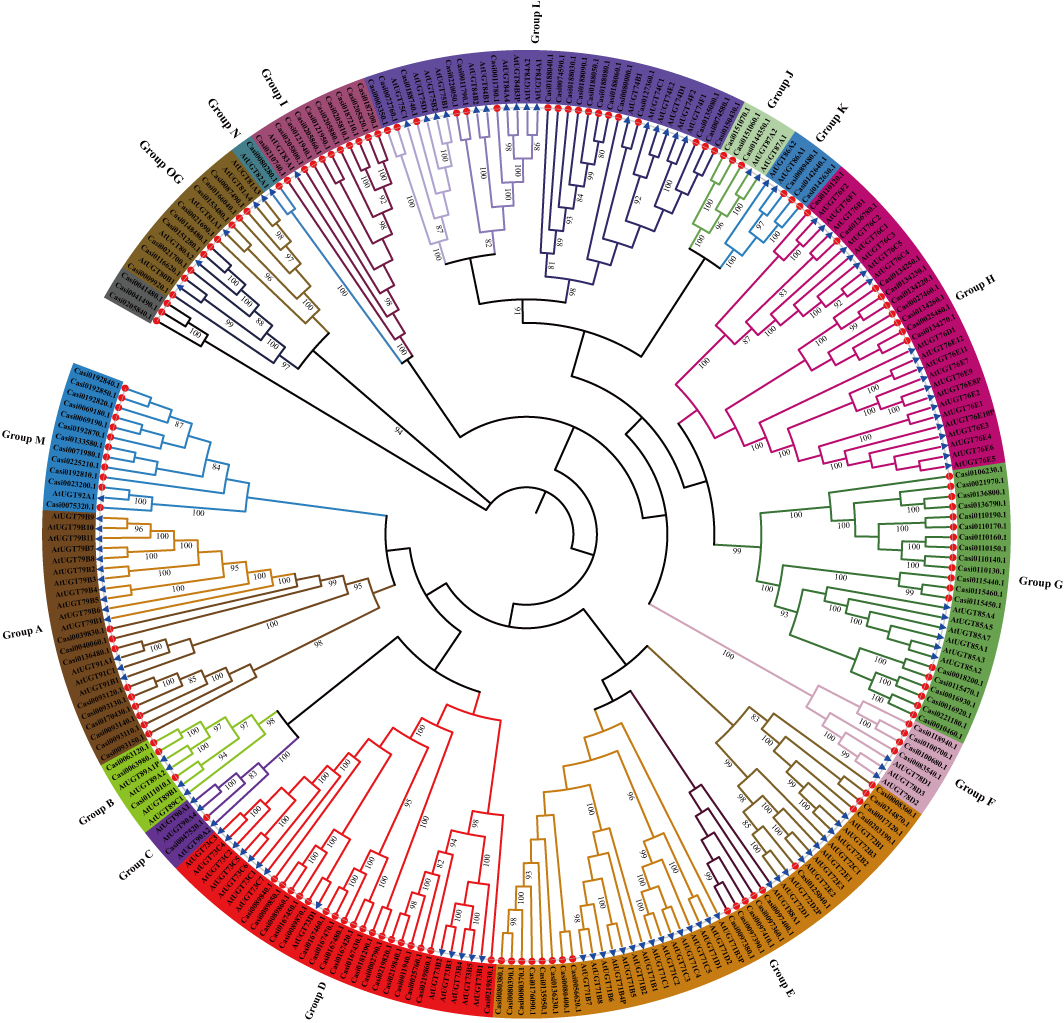

Supplement: Supplementary Figure 9 — The phylogenetic tree of UGTs from C. asiatica and Arabidopsis. The representative UGT family members from C. asiatica and Arabidopsis are marked with red circles and blue triangles, respectively. [file Image_9.jpeg]
